# Differential expression of enzymes in thymidylate biosynthesis in zebrafish at different developmental stages: implications for *dtymk* mutation-caused neurodegenerative disorders

**DOI:** 10.1186/s12868-022-00704-0

**Published:** 2022-03-27

**Authors:** Junmei Hu Frisk, Stefan Örn, Gunnar Pejler, Staffan Eriksson, Liya Wang

**Affiliations:** 1 Department of Anatomy, Physiology and Biochemistry, Swedish University of Agricultural Sciences, Uppsala, Sweden; 2 Department of Biomedical Sciences and Veterinary Public Health, Swedish University of Agricultural Sciences, Uppsala, Sweden; 3 Department of Medical Biochemistry and Microbiology, Uppsala University, Uppsala, Sweden

**Keywords:** Thymidylate kinase, *Dtymk*, Thymidine kinase, *Tk*, dTTP synthesis, dNTPs, Zebrafish development, Neuronal development

## Abstract

**Background:**

Deoxythymidine triphosphate (dTTP) is an essential building block of DNA, and defects in enzymes involved in dTTP synthesis cause neurodegenerative disorders. For instance, mutations in *DTYMK*, the gene coding for thymidylate kinase (TMPK), cause severe microcephaly in human. However, the mechanism behind this is not well-understood. Here we used the zebrafish model and studied (i) TMPK, an enzyme required for both the de novo and the salvage pathways of dTTP synthesis, and (ii) thymidine kinases (TK) of the salvage pathway in order to understand their role in neuropathology.

**Results:**

Our findings reveal that maternal-stored dNTPs are only sufficient for 6 cell division cycles, and the levels of dNTPs are inversely correlated to cell cycle length during early embryogenesis. TMPK and TK activities are prominent in the cytosol of embryos, larvae and adult fish and brain contains the highest TMPK activity. During early development, TMPK activity increased gradually from 6 hpf and a profound increase was observed at 72 hpf, and TMPK activity reached its maximal level at 96 hpf, and remained at high level until 144 hpf. The expression of *dtymk* encoded Dtymk protein correlated to its mRNA expression and neuronal development but not to the TMPK activity detected. However, despite the high TMPK activity detected at later stages of development, the Dtymk protein was undetectable. Furthermore, the TMPK enzyme detected at later stages showed similar biochemical properties as the Dtymk enzyme but was not recognized by the Dtymk specific antibody.

**Conclusions:**

Our results suggest that active dNTP synthesis in early embryogenesis is vital and that Dtymk is essential for neurodevelopment, which is supported by a recent study of *dtymk* knockout zebrafish with neurological disorder and lethal outcomes. Furthermore, there is a novel TMPK-like enzyme expressed at later stages of development.

**Supplementary Information:**

The online version contains supplementary material available at 10.1186/s12868-022-00704-0.

## Background

Zebrafish (*Danio rerio*) is a useful vertebrate model organism because of the external fertilization and fast embryonic development with a high level of transparency, which provides a unique opportunity for biomedical and genetic studies. The rapid generation of genetically modified zebrafish lines by Crispr/Cas9 or morpholino approaches allows detailed analyses of gene/protein functions in a vertebrate context. The zebrafish genome-sequencing project reveals that approximately 70% of the human genes have zebrafish orthologues [1]. Therefore, the zebrafish model has contributed profoundly to our knowledge of vertebrate genetics and biology [2–4].

Nucleotides are essential components of cellular metabolic processes and are synthesized by two distinct pathways - the de novo and salvage pathways. Deoxythymidine triphosphate (dTTP) is an essential building block of DNA and can be synthesized by the de novo pathway where deoxyuridine monophosphate is converted to thymidine monophosphate (dTMP) by thymidylate synthase in the presence of tetrahydrofolate, or by the salvage pathway where deoxythymidine (dThd) is phosphorylated to dTMP by either cytosolic thymidine kinase 1 (TK1) or mitochondrial thymidine kinase 2 (TK2). dTMP, i.e., produced either by the de novo or salvage pathway, is then further phosphorylated to dTTP in two consecutive steps catalyzed by thymidylate kinase (TMPK) and the non-specific nucleoside diphosphate kinases. TMPK is thus an enzyme required for both the salvage and de novo pathways of dTTP synthesis [5–7].

A balanced deoxynucleoside triphosphates (dNTPs) pool is essential for DNA synthesis, repair and cell proliferation. Therefore, enzymes involved in nucleotide synthesis play important roles in cell proliferation and survival. Loss of function mutations or dysregulation of key enzymes in nucleotide metabolism can lead to severe pathophysiological conditions, including neurological and myopathic disorders, such as neonatal liver failure, nystagmus, hypotonia, and growth retardation [8]. In humans, deficiency in TK2 activity due to genetic alterations causes devastating mitochondrial DNA depletion and/or deletion diseases affecting multiple tissues [6, 9]. Genetic alterations of the *DTYMK* gene, coding for TMPK, cause severe neurodegenerative disorders [10, 11], suggesting that TMPK is essential for neuronal development.

In the recent two decades, using different genetic approaches, a large number of genes coding for enzymes in nucleotide metabolism have been studied in zebrafish [2, 12–15]. For example, mutations in *gart* and *paics* in the de novo purine biosynthesis cause pigmentation and ocular developmental disorders [14]; mutations in *cad*, encoding carbamoyl-phosphate synthetase 2 - aspartate transcarbamoylase - dihydroorotase in pyrimidine de novo synthesis, lead to severe dismorphogenesis of the jaw and fin, and to a small eye phenotype; and mutations in ribonucleotide reductase, an essential enzyme for the de novo dNTP synthesis, result in severe deficits and early embryonic lethality [13].

To understand the essential role of enzymes in dTTP synthesis in development, we used zebrafish as a model to study the expression and activity of TMPK and TK enzymes at different developmental stages. Our findings suggested the existence of two TMPK enzymes, which apparently were differentially expressed. Further, we demonstrated that the expression and activity of salvage pathway enzymes, i.e., TK1 and TK2, were low and probably insignificant for dTTP synthesis during early embryogenesis. Our results also showed that dNTP levels were inversely correlated to cell cycle length during early embryogenesis, thus representing limiting factors for cell division in zebrafish.

## Results

### dNTP levels are inversely correlated to cell cycle length during early embryogenesis

**Fig. 1:**
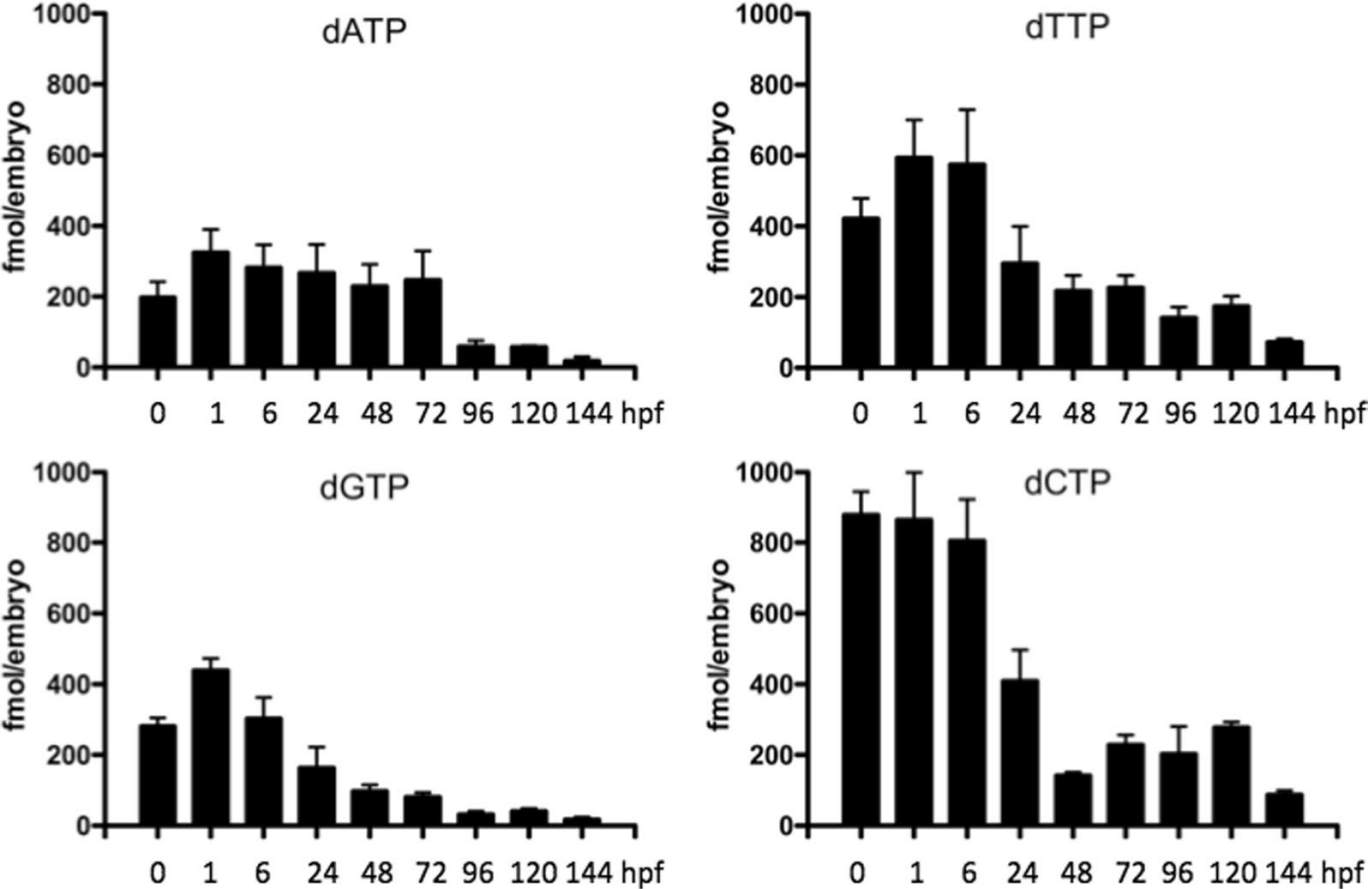
The levels of dNTPs at different developmental stages. A total of 50–75 embryos/larvae were collected at each time point and used in dNTP measurement (see Materials and Methods for details). Data were from 9 to 12 independent measurements and given as fmol dNTP per embryo (mean ± SD)

Zebrafish embryos undergo rapid cell division during early stages of development. By the time zygotic genome activation starts, approximately 3.5 hpf, the embryos have gone through 12 cycles of cell division with approximately 15 to 30 min per cycle [16]. This rapid cell division requires adequate supply of dNTPs for nuclear DNA synthesis. Here we determined the levels of dNTPs at different developmental stages. As shown in Fig. 1, the overall levels of dCTP and dTTP were higher than those of dATP and dGTP during the entire measurement period, and the highest dNTP levels were maintained from 0 to 6 hpf and then started to decline gradually to a low level at 144 hpf. These results suggest that there is an active dNTP synthesis during the first 6 h after fertilization, probably by maternal supplied essential enzymes and mRNA transcripts encoding dNTP synthesis enzymes before zygotic genome activation. The zebrafish genome contains 1.41 × 10^9^ base pairs with an AT content of 61.4%. Using the dNTP concentrations determined at 0 hpf and method described by Song et al. [17], we could conclude that the embryo contained at least 70 nuclear equivalents of dATP, which was sufficient for 6 rounds of nuclear division, and the levels of dTTP, dGTP and dCTP could sustain even higher numbers of cell divisions (Fig. 1). Until the 10th cell division (3 hpf) each cell cycle needs only 15–20 min, and the high levels of dNTPs measured here from 0 to 6 hpf ensured that there are sufficient dNTPs to sustain rapid cell growth. From 6 hpf and onward, the dNTP levels decreased gradually to their lowest levels at 144 hpf. Similarly, from cell division cycle 12 and onwards, the time required for each cell division gets progressively longer, with 240 min needed for the 16th cell cycle [16]. These results indicated that the levels of dNTPs are limiting factors during cell division and that levels of dNTPs inversely correlate with the time required for each cell cycle.

### High TMPK activity in developing zebrafish embryos

**Fig. 2:**
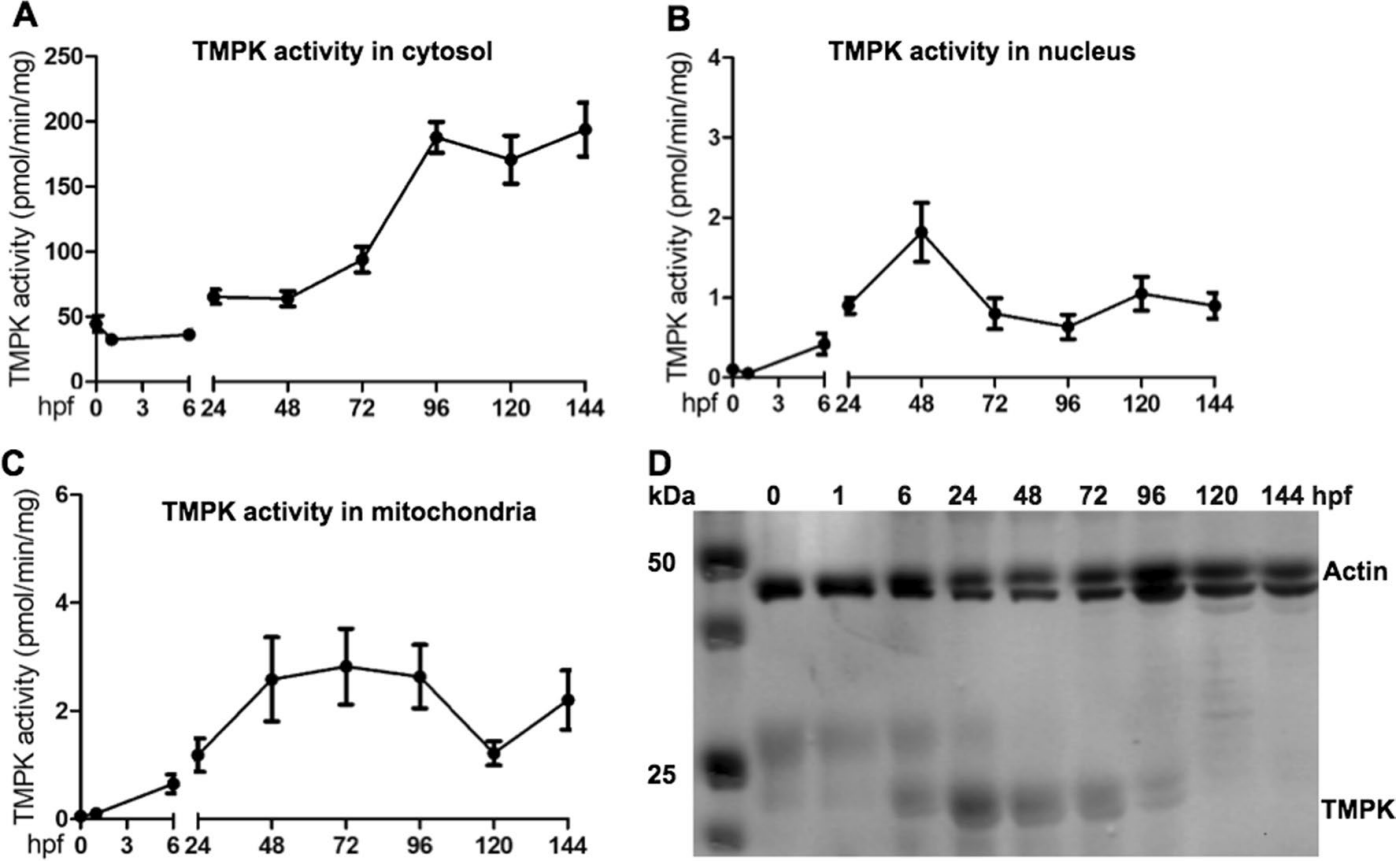
The levels of TMPK at different developmental stages. TMPK activity in the cytosol (**A**), nucleus (**B**) and mitochondria (**C**). **D** TMPK protein levels in cytosolic fractions. Cytosolic, mitochondrial and nuclear fractions were isolated from zebrafish at 0, 6, 24, 48, 72, 96, 120 and 144 h post fertilization (hpf). Total protein was extracted from these fractions and used to measure TMPK activity by using [^3^ H]-dTMP as substrate and the data was plotted as mean ± SD. TMPK protein levels was assessed by western blot analysis using a specific antibody designed against zebrafish canonical TMPK and only the relevant part of the full image is shown

TMPK is a key enzyme in dTTP synthesis both for the de novo and the salvage pathways. Therefore, we studied zebrafish TMPK activity in embryos and larvae harvested at different developmental stages. The embryos/larvae were homogenized and fractionized directly into cytosolic, nuclear, and mitochondrial fractions. Total protein from these fractions was extracted and used to measure TMPK activity using tritium labeled dTMP as substrate. TMPK activity was detected mostly in the cytosolic compartment (Fig. 2A), while in mitochondrial and nuclear fractions TMPK activities were very low, only approximately 1–5% of the TMPK activity detected in the cytosol (Fig. 2B and C). As shown in Fig. 2A, directly after fertilization the cytosolic TMPK activity was at basal level and slightly decreased in comparison with the zero time point, but then started to increase, reaching a maximum at 72 hpf, after which the TMPK activity was profoundly increased; this coincided with the hatching point. The highest TMPK activity was reached at 96 hpf and TMPK activity was maintained at this high level throughout the studied time frame (up to 144 hpf) (Fig. 2A).

### Two TMPK proteins are differentially expressed at different developmental stages

Using a specific antibody raised against the active site region of the *dtymk*-encoded TMPK (hereafter referred to as the “canonical Dtymk”), the canonical Dtymk protein levels were determined. Notably, the western blot analysis revealed a fundamentally different pattern of the canonical Dtymk protein expression in comparison with the TMPK activity profile shown in Fig. 2A; low levels of the canonical Dtymk protein were detected at 0 and 1 hpf, thereafter, it started to increase from 6 hpf and reached its maximum at 24 hpf, after which it decreased gradually down to being barely detectable at 96 hpf. From 120 hpf and onwards the canonical Dtymk protein was undetectable (Fig. 2D). These findings suggest that the high TMPK activity detected from 96 to 144 hpf (see Fig. 2A) is most likely not attributed by the canonical Dtymk protein, hence introducing the notion that an alternative non-canonical TMPK like enzyme is expressed at high levels at later stages of development. This non-canonical TMPK is most likely encoded by an unknown gene, since in the zebrafish genome there is only one known TMPK-encoding gene: *dtymk*.

### Biochemical properties of the zebrafish TMPKs

**Fig. 3:**
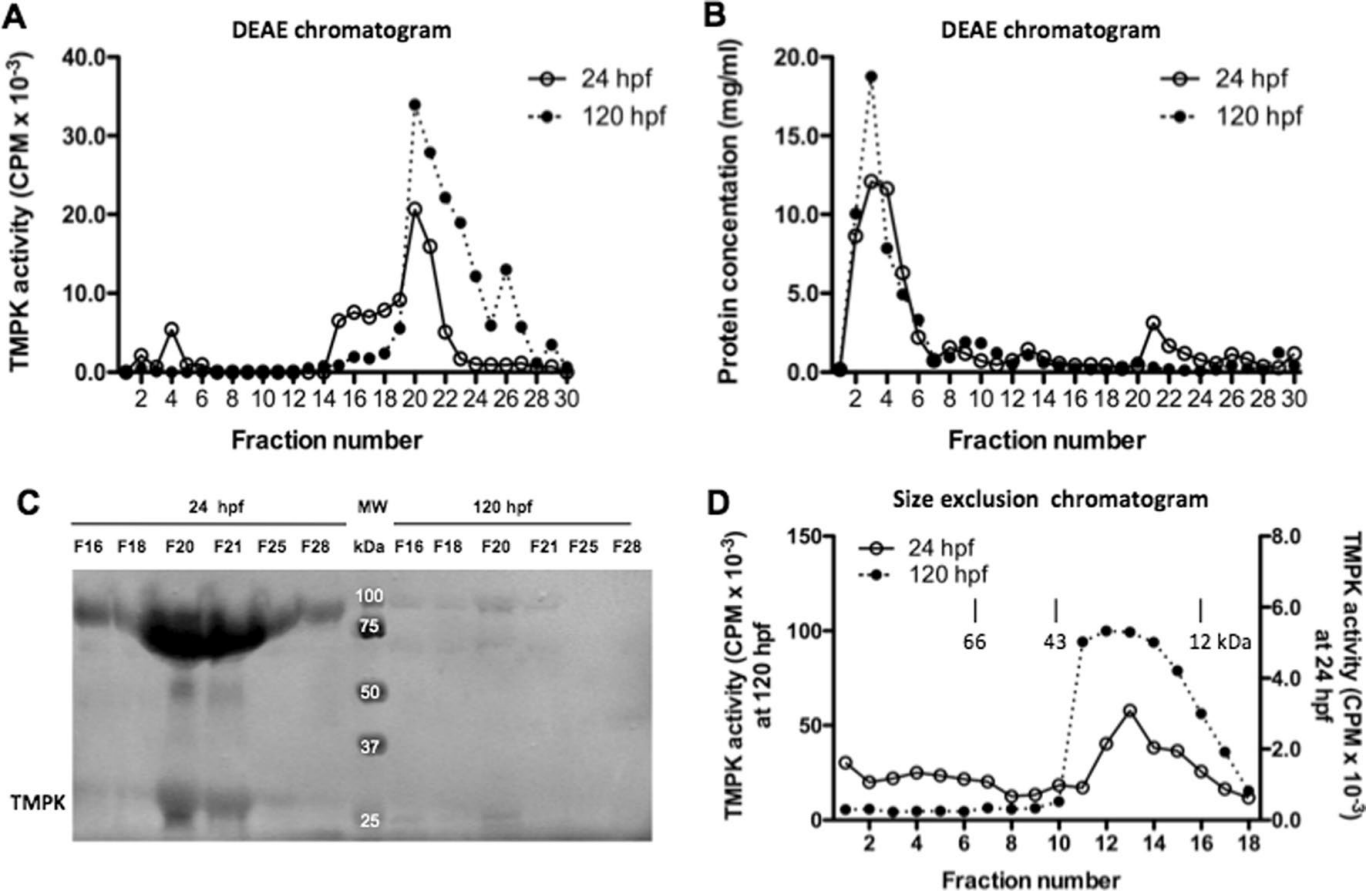
Characterization of cytosolic TMPK. **A** Partial purification of zebrafish TMPK by DEAE chromatography using cytosolic proteins isolated from zebrafish embryos harvested at 24 hpf and 120 hpf. The bound proteins were eluted with stepwise increase of KCl concentration (from 50 to 250 mM) in DEAE buffer. Fractions were collected and assayed for TMPK activity; **B** Protein concentration of the corresponding DEAE fractions; **C** Identification of the canonical TMPK proteins in the TMPK activity peak fractions by western blot analysis using a zebrafish Dtymk-specific antibody. Fractions F16, F18, F20, F21, F25, and F28 from DEAE chromatography were used and only part of the image is shown; **D** Size exclusion chromatography of partially purified zebrafish TMPK. Fractions F20 and F21 from the DEAE chromatography were used. Fractions were collected and assayed for TMPK activity. The Y-axis on the left side corresponded the TMPK activities from 120 hpf embryos and the Y-axis on the right side corresponded the TMPK activities from 24 hpf embryos. The elution positions of molecular weight markers are indicated

Next, we analyzed the cytosolic TMPK proteins by using anion exchange chromatography. For this, we used TMPK protein isolated from embryos harvested at 24 hpf, a stage at which the canonical Dtymk protein was expressed at its highest level, and from larvae at 120 hpf at which the canonical Dtymk protein was undetectable. Total cytosolic proteins were loaded onto DEAE columns, followed by elution with stepwise increased salt concentrations. Fractions were collected and TMPK activity determined. As shown in Fig. 3A, the major TMPK activity that bound to the column was eluted with 200 mM KCl, with the highest TMPK activity observed in fraction 20 and 21 for proteins isolated from embryos/larvae harvested both at 24 and 120 hpf. The protein concentration in each fraction was also determined (Fig. 3B). Fractions with TMPK activity (fraction number 16, 18, 20, 21, 25 and 28) were then analyzed by western blot using the antibody against the canonical Dtymk protein. As shown in Fig. 3C, the canonical Dtymk protein was detected in fractions with TMPK activity isolated from embryos harvested at 24 hpf, with the highest TMPK protein levels seen in fraction 20 and 21, which coincided with the TMPK activity as shown in Fig. 3A. In addition to the ~ 25 kDa band representing monomeric Dtymk protein, we also noted a band of ~ 75 kDa. Most likely, this band corresponds to oligomerized Dtymk. However, for proteins isolated from larvae harvested at 120 hpf, the canonical Dtymk protein was below the level of detection in all of the fractions containing TMPK activity (Fig. 3C). Hence, these findings are consistent with the results shown in Fig. 2D, providing support for the existence of two different TMPK enzymes, expressed at 24 and 120 hpf embryos/larvae, respectively.

We further analyzed the partially purified TMPKs by size exclusion chromatography to determine their molecular weight (MW). TMPK from embryos harvested at 24 hpf eluted with a MW of ~ 20–30 kDa, and the TMPK from larvae harvested at 120 hpf eluted at a similar MW (Fig. 3D). Thus, the TMPK proteins presented in the cytosol isolated from 24 to 120 hpf embryos/larvae apparently have similar biochemical properties and molecular weight but clearly different immuno-reactivity towards antibody against the canonical Dtymk.

### Tk2 is the dominant cytosolic Tk expressed in zebrafish

**Fig. 4:**
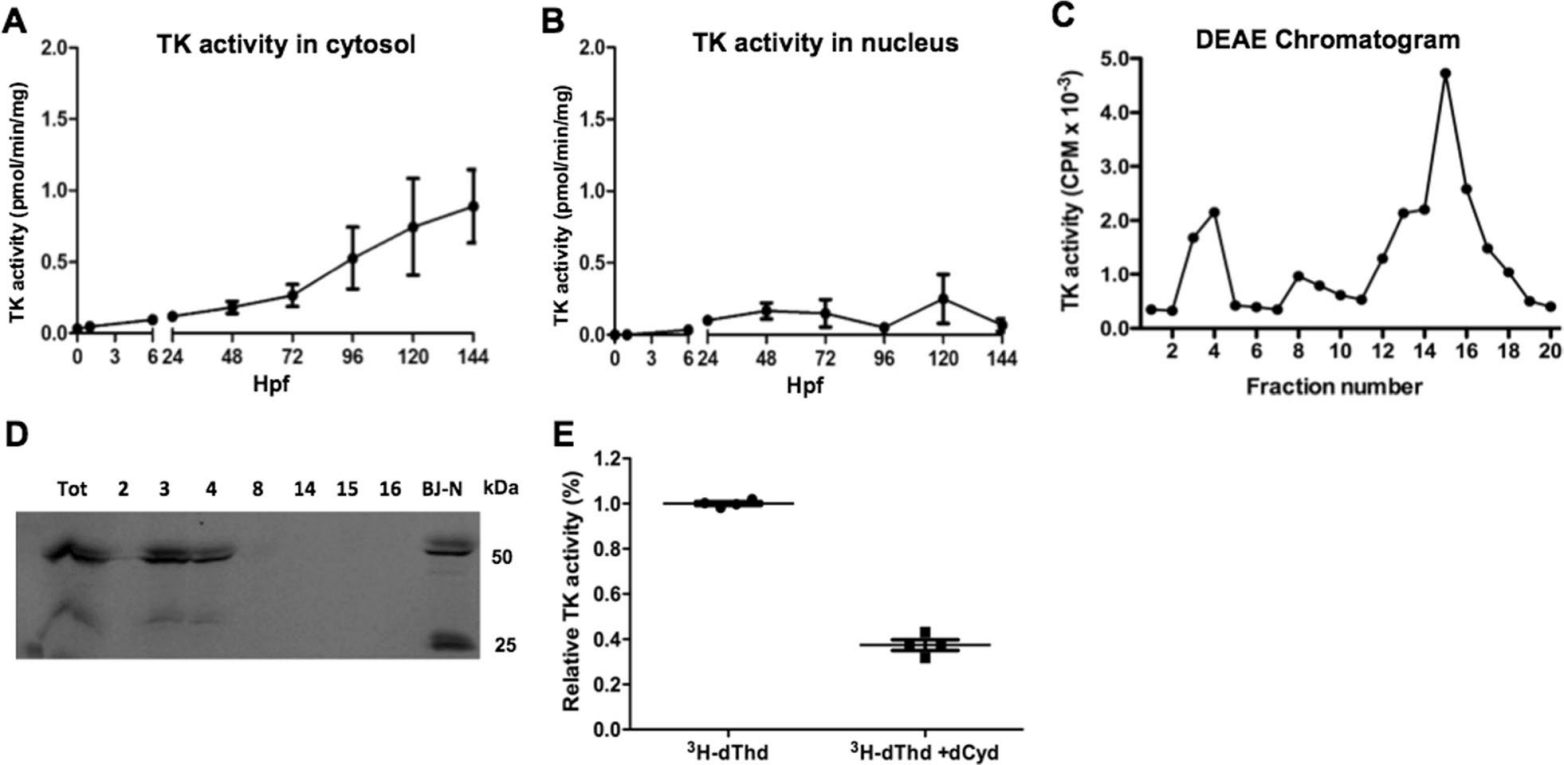
Characterization of thymidine kinases. TK activity in the cytosol (**A**) and nucleus (**B**) fractions at different zebrafish developmental stages. **C** DEAE chromatography of cytosolic protein isolated from zebrafish at 120 hpf. Fractions were collected and TK activity was determined by using [^3^ H]-dThd as substrate. **D** Western blot analysis of the TK peak fractions (2, 3, 4, 8, 14,15 and 16) from DEAE chromatography using a TK1 specific antibody and only part of the image is shown. Cell extracts from a fibroblast (BJ-N) cell line was used as control. **E** Fractions from DEAE chromatography (fraction 15 and 16) were assayed in the absence and presence of excess dCyd using [^3^ H]-dThd as the substrate. Data was plotted as mean ± SD

The contribution of the salvage pathway synthesis of dTTP was studied by measuring Tk activity in embryos and larvae harvested at different developmental stages. In contrast to the high cytosolic TMPK activity during embryonic development, Tk activity in the cytosolic compartment was very low at early time points, whereafter it increased gradually with time and reached the highest levels at 144 hpf (Fig. 4A). The Tk activity in the nuclear fraction was even lower, approximately half of that in the cytosol (Fig. 4B), whereas no Tk activity was detected in the mitochondrial fractions (data not shown). Notably, this is in contrast to TKs from other species, where high TK activity is present in the cytosol, and lower TK activity is present in mitochondrial and nuclear fractions from rapidly dividing cells [5, 18].

We next analyzed the cytosolic proteins by DEAE column chromatography. As shown in Fig. 4C, two major Tk activity peaks were identified, with the first peak eluting at low salt (fraction 3–4) and the second peak eluting in the high salt fractions (fraction 14–16). Western blot analysis of the Tk activity peak fractions using a Tk1-specific antibody showed the presence of Tk1 protein in the fractions corresponding to the Tk activity eluting with low salt during DEAE chromatography, whereas no Tk1 protein was detected in fractions eluting with high salt (Fig. 4D). When fractions 14–16 (corresponding to the high salt peak of Tk activity) were assayed with ^3^ H-dThd in the presence of excess of deoxycytidine (dCyd), Tk activity was reduced by 65% (Fig. 4E). This indicates that the Tk activity recovered in the high salt peak corresponds to Tk2, since Tk2 uses both dThd and dCyd as substrates whereas Tk1 phosphorylates only dThd [5].

## Organ distribution of TMPKs and TK in zebrafish

**Fig. 5:**
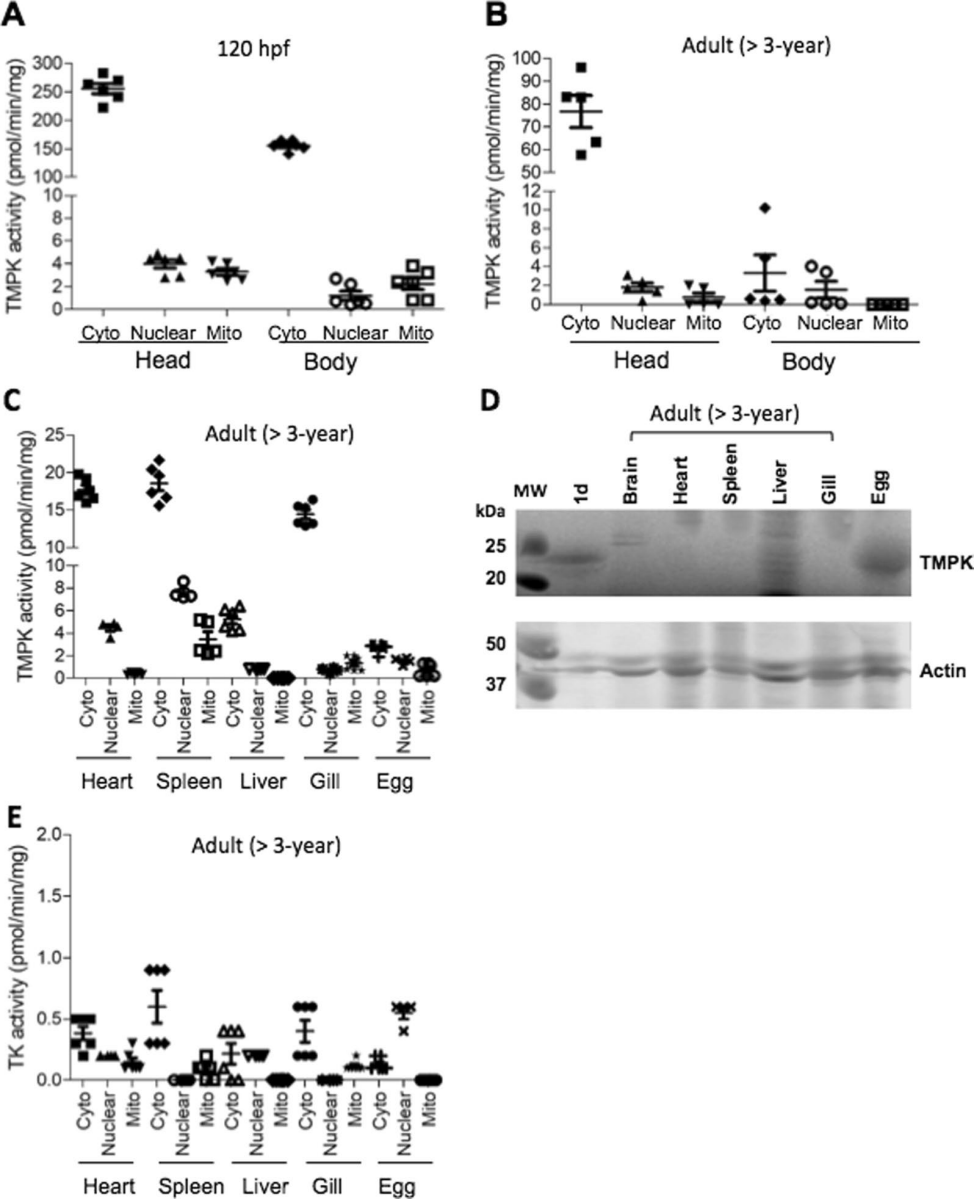
Organ distribution of TMPK and TK. TMPK activity in larvae at 120 hpf (**A**) and adult fish (> 3 years old) (**B** and **C**). **D** TMPK protein levels in different organs. **E** TK activity in different organs. The head and truncated body (without head) as well as organs from adult fish were used to isolate cytosolic, mitochondrial and nuclear fractions. Total proteins were extracted and used to measure TMPK and TK activity by using [^3^ H]-labeled substrates and the data was plotted as mean ± SD. Western blot analysis was conducted by using the zebrafish Dtymk-specific antibody and only parts of the images are shown

TMPK and TK1 are known to be cell cycle regulated with the highest expression levels in proliferating cells and tissues [19, 20]. Neurons are usually regarded as post-mitotic without the ability to re-enter the cell cycle [21], and therefore, it is conceivable to assume that TMPK and TK1 activity would be low. Here, we examined tissue distribution of TMPK and TK activity in zebrafish larvae at 120 hpf and adult fish (> 3 years old). The cytosolic, mitochondrial and nuclear TMPK activity in the zebrafish head were compared with the remainder of the body (decapitated zebrafish) from zebrafish larvae at 120 hpf and adult fish (> 3 years old), as well as in different organs from adult fish. Similar to the results shown in Fig. 2, the highest TMPK activity was detected in the cytosolic fractions of all samples and the levels of TMPK activity in the nuclear and mitochondrial fractions were very low (Fig. 5A–C). In zebrafish larvae from 120 hpf, TMPK activity was much higher in the head than the remainder of the body, and a similar pattern was also observed in adult fish (Fig. 5A, B). Compared with adult fish, brain TMPK activity from zebrafish larvae at 120 hpf was 3 times higher, and the remaining body of zebrafish larvae at 120 hpf contained 50 times higher TMPK activity (Fig. 5A and B). In other organs from adult fish, the specific TMPK activity was more than 4 times lower as compared with the brain. Furthermore, heart, spleen and gill had higher TMPK activity levels than liver and unfertilized egg (Fig. 5C).

Next, we analyzed the levels of the canonical Dtymk protein in different organs from adult fish by using western blot analysis, and found that the canonical Dtymk protein was not detectable in any of the organs from adult fish, while in the 24 hpf embryos and unfertilized egg the canonical Dtymk protein could be detected (Fig. 5D). These results indicate that the canonical Dtymk is mainly expressed during early embryogenesis and that the alternative non-canonical TMPK enzyme is expressed in adult fish.

TK activity in different organs was also measured. In agreement with the data displayed in Fig. 4, TK activities were higher in the cytosolic fractions recovered from different organs, in comparison with TK activities in mitochondrial and nuclear fractions. However, the overall TK activity was very low in all tested organs (Fig. 5E).

## The mRNA expression profile of enzymes in thymidine nucleotide biosynthesis

**Fig. 6:**
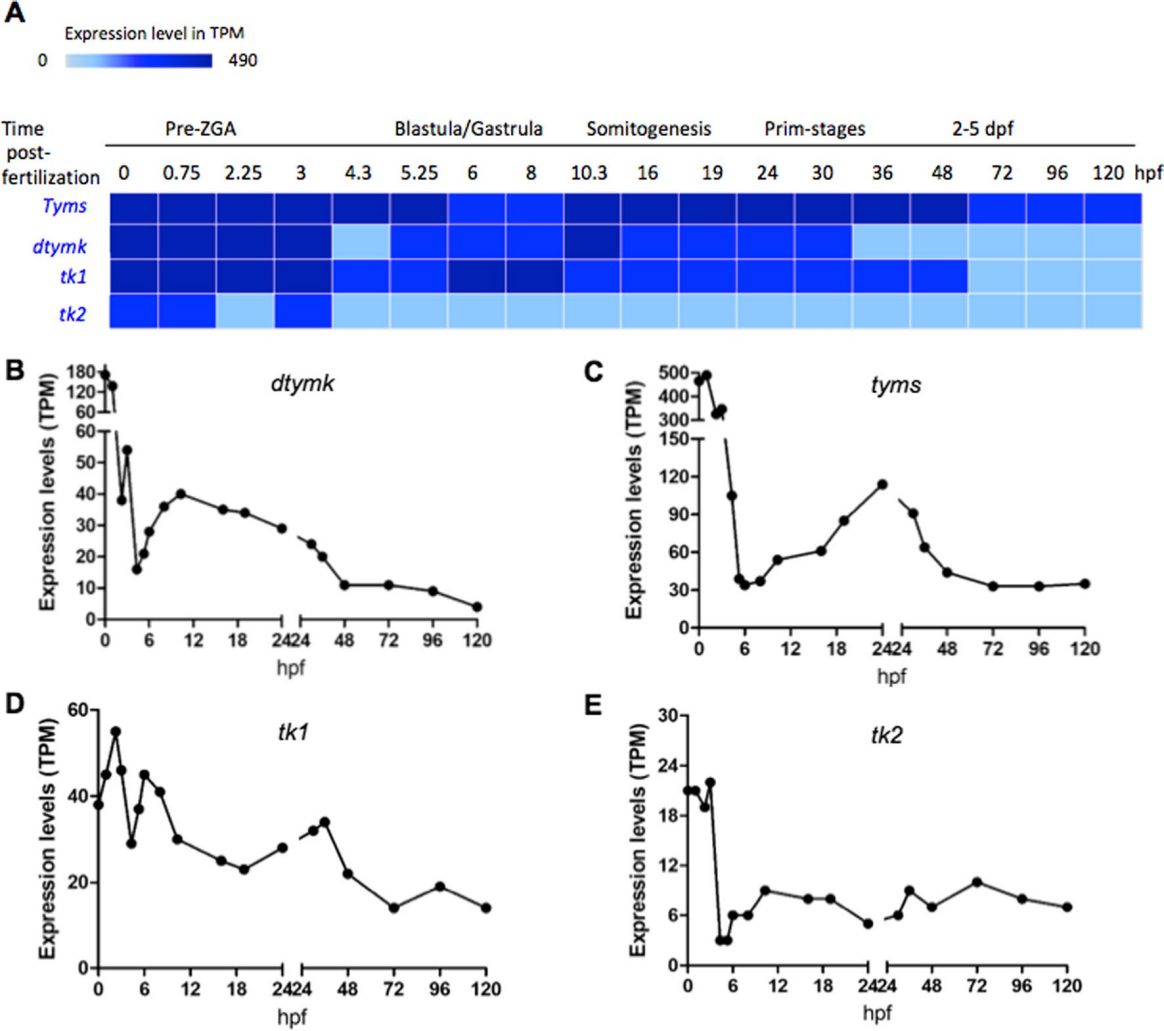
mRNA expression profiles of selected enzymes involved in dTTP synthesis at different zebrafish developmental stages. **A** mRNA expression heatmap. *dtymk*, thymidylate kinase; *tyms*, thymidylate synthesis; *tk1*, thymidine kinase 1; *tk2*, thymidine kinase 2.  Plots of mRNA levels of *dtymk* (**B**), *tyms* (**C**), *tk1* (**D**) and *tk2* (**E**). TPM, transcript per million. Data are shown as average expression (mean TPM) and retrieved from Expression atlas – baseline expression of transcriptional profiling of zebrafish developmental stages (https://www.ebi.ac.uk/gxa/experiments/E-ERAD-475/Result)

We next assessed the mRNA expression profiles of enzymes involved in thymidine nucleotide biosynthesis: *dtymk* (coding for the canonical Dtymk), *tyms* (coding for Tyms), *tk1* (coding for Tk1) and *tk2* (coding for Tk2) during different developmental stages, using data available at the Expression Atlas database (http://www.ebi.ac.uk/gxa/experiments/E-ERAD-475) [22]. As shown in Fig. 6, the expression of *tyms* mRNA is the highest of all four genes included in our analysis, and *dtymk* mRNA levels is lower than that of *tyms* mRNA but higher than the levels of *tk1* and *tk2* mRNA. Prior to zygotic genome activation (pre-ZGA), the levels of *tyms, dtymk, tk1 and tk2* mRNA are relatively high and then decline to baseline levels at 6 hpf. After zygotic genome activation, the *dtymk* mRNA levels increase again during blastula/gastrula stages and reach its peak levels at approximately 10 hpf and then decline to baseline levels at 48 hpf, remaining at low levels onwards (Fig. 6B). Similar to *dtymk*, the level of *tyms* mRNA also increased after zygotic genome activation and reaches a peak level during somitogenesis (24 hpf), and then declines to baseline at 72 hpf and remains at the same level during the study time frame (Fig. 6C). The levels of *tk1* and *tk2* mRNA are low as compared with those of *dtymk* and *tyms*, and fluctuate during the different developmental stages (Fig. 6D, E).

## Discussion

An adequate supply of dNTPs is essential for DNA replication and repair, and thus, cell proliferation. Early studies have shown that maternal-stored dNTPs is only sufficient for a limited number of cell divisions during early embryogenesis, for example, 11 rounds of cell division in *Xenopus* and 12 division cycles in *Drosophila* [17, 23]. Therefore, embryos must produce their own dNTPs in early stages in order to cope with rapid cell division during embryogenesis. Our data show that, in zebrafish embryo, the maternal-stored dNTP is only sufficient for 6 rounds of cell division and that the dNTP synthesis capacity is high in zebrafish embryos to sustain repaid cell division. Furthermore, the levels of dNTP detected at different developmental stages inversely correlate with cell cycle length [16], demonstrating that dNTP levels are limiting factors for cell proliferation.

There are four nucleoside monophosphate kinases, i.e. adenylate kinases, guanylate kinase, cytidylate-uridylate kinases, and TMPK, which catalyze the phosphorylation of nucleoside monophosphates to their corresponding nucleoside diphosphates. These enzymes have very narrow substrate specificity, and TMPK is the only nucleoside monophosphate kinase that is known to be able to phosphorylate dTMP [24]. Therefore, TMPK is essential for dTTP biosynthesis and thereby plays a critical role in cell growth. Our study demonstrated that in zebrafish TMPK activity is mainly present in the cytosol with very low activity in mitochondria and nucleus, irrespective of developmental stages or organ/tissue origin. This cytosolic distribution of zebrafish TMPK is similar to what has been observed in other species [24–26]. We also showed that, during early embryonic development, TMPK activity was at a relatively low but stable level (from 0 to 6 hpf), which is sufficient to sustain an adequate dTTP pool for rapid DNA synthesis and cell division. After activation of the zygotic genome, from 6 to 24 hpf, TMPK activity increased gradually and a drastic increase in TMPK activity was seen after 72 hpf, i.e. at a stage where zebrafish switch from the embryonic to larval stage, from which cell growth escalates [27]. Hence, this marked increase in TMPK activity is consistent with the high demand for thymidine nucleotides at this phase of development.

We also compared TMPK activity in different organs and found that brain contained the highest TMPK activity, both at the larval stage (120 hpf) and in adult fish (> 3 years). Furthermore, the overall TMPK activity was much higher at the larval stage than in adult fish, and in different organs from adult fish the TMPK activity level were in the order of brain > heart > spleen > gill > liver.

An intriguing finding in this study was the presence of high TMPK activity that may not be attributed by the canonical Dtymk protein (encoded by *dtymk*). This non-canonical TMPK activity appeared to be expressed from ~ 96 hpf and onwards into adulthood. Notably, at 120–144 hpf and also in adult fish, the canonical Dtymk protein was undetectable by Western blot analysis using a Dtymk specific antibody despite the high TMPK activity detected. Notably, the Dtymk antibody was raised against a 13-amino acid peptide sequence that is localized within the Lid region of human TMPK sequence, which is an essential structural element for enzyme catalysis [28]. Hence, an alternatively spliced isoform lacking this sequence would not fold into an active enzyme, and the failure of the antibody to detect any alternative variants of Dtymk suggests that enzymatically active, alternatively spliced isoforms of Dtymk are not expressed in the zebrafish. Together, these results provide support for the existence of an alternative non-canonical TMPK gene in zebrafish. Along the same line, we noted that the canonical *dtymk* mRNA expression was high at earlier stages of embryonic development but dropped to low levels at later stages (from 48 hpf and onwards), which is not correlated with the TMPK activity levels. These results also support the presence of an alternative TMPK gene.

In fibroblasts derived from a human patient with loss of function mutations in the *DTYMK* gene, the cells grew normally and level of dTTP showed no significant difference from that of the controls, and the loss of function mutations affected only the brain but not other organs [11, 29], suggesting the presence of a compensatory TMPK like enzyme expressed in other organs. Furthermore, in a human fibroblast cell line, the existence of an alternative TMPK enzyme has recently been described [30]. Our results show that the non-canonical TMPK activity detected at late stages had similar biochemical properties as the canonical Dtymk protein regarding isoelectric point and molecular weight, whereas it differs in not being recognized by antibody raised against the canonical Dtymk. However, the identity of this potential non-canonical TMPK, either in zebrafish or in humans is presently not known, but represents an important task for further investigations.

Zebrafish embryos start their morphogenesis at 4.5 hpf, and the first neurons become post-mitotic in the neural plate shortly after gastrulation (5.5 hpf). The time between 10 and 24 hpf is a critical time period when the embryo’s primary organs start to develop, the nervous system is further expanded, and the brain is further developed at the end of the first day (24 hpf). During the next two days (48 and 72 hpf), the completion of the primary organ system is reached [31–33]. The expression pattern of the canonical Dtymk protein showed a close correlation with organogenesis and neuronal development; i.e. the levels of the canonical Dtymk protein increased significantly at 6 hpf and reached its maximum at 24 hpf, and then declined gradually until 72 hpf, a time point before the expression of the non-canonical TMPK activity starts to increase. These results suggest that the canonical Dtymk protein may play an important role in neurodevelopment, and that deficiency in TMPK activity caused by mutations in the *dtymk* gene would preferentially affect nervous system development. As shown in a recent study where *dtymk* knockout in zebrafish resulted in microcephaly, neuronal cell death and early lethality [11].

In the zebrafish genome database, one *tk1* transcript (GenBank: AAO64437.1) and two *tk2* transcripts (GenBank: NP_001002743.2 and AAH76441.1) were reported. The two *tk2* transcripts differ mainly in their N-terminal sequences: one (NP_001002743.2) with a putative mitochondrial targeting signal sequence, which is lacking in the second transcript (AAH76441.1), and thus, the second transcript is most likely encoding a cytosolic protein. Thus, the first TK activity peak seen after DEAE chromatography corresponds to Tk1, which was also confirmed by western blot analysis using a Tk1 specific antibody, and the second TK activity peak corresponded to the cytosolic form of Tk2, which was also confirmed by TK activity assay in the presence of excess of dCyd. Altogether, these results suggest that Tk2 is the prominent TK present in the cytosol of zebrafish cells.

Thymidylate synthase (Tyms) is also a key enzyme in the de novo dTTP synthesis pathway and is expressed throughout all of the developmental stages, with the highest levels of Tyms protein detected at 1–4 cell (0.2 to 1 hpf) stage [34], and the levels of Tyms protein correlate with its mRNA expression profile [22]. Knockdown of *tyms* by siRNA technique or insertion mutagenesis resulted in a serious delay in tail and head development [13, 34]. However, thymidine supplement could reverse the abnormality observed in *tyms*-knockdown zebrafish [34]. These results suggest that dTTP is a limiting factor in zebrafish development and that the salvage pathway of dTTP synthesis through TK-catalyzed reactions can compensate for the defect in the de novo dTTP synthesis pathway caused by *tyms*-knockdown if sufficient deoxynucleosides are available, although the levels of Tk1 and Tk2 activity and mRNA are low.

In cultured cells the expression of the canonical TMPK is cell cycle-regulated with the highest level during the S-phase, and TMPK activity levels correlated with cell growth rates. In non-proliferating cells/tissues, the canonical TMPK activity is very low [19]. In yeast, defects in dTTP synthesis due to mutations in either *cdc8* (coding for TMPK) or *cdc21* (coding for Tyms) leads to telomere shortening and growth arrest [35]. In humans, mutations of the canonical TMPK coding gene (*DTYMK*) causes severe neurodegenerative disorders, but have no clear effects on other organs, nor on overall metabolism in the affected patients [10, 11]. Together, these results suggest that *DTYMK* is required for neuronal development during early embryogenesis, whereas the putative non-canonical TMPK is expressed at a later stage of development. Future studies of the canonical TMPK and identification of the putative non-canonical TMPK may help to elucidate the mechanism by which TMPK deficiency leads to neurodegenerative disorders.

## Conclusions

Our results suggest that active dNTP synthesis in early embryogenesis is vital. TMPK and TK activities are prominent in the cytosol of embryos, larvae and adult fish and brain contains the highest TMPK activity. The expression of the *dtymk*-encoded Dtymk protein is correlated to neurodevelopment but not to the TMPK activity levels detected at late stages of development. These results indicate that Dtymk is essential for neurodevelopment, which is supported by a recent study of *dtymk* knockout zebrafish with neurological disorders and fatal outcomes [11]. The TMPK-like enzyme expressed at later stage of development showed similar biochemical properties as the Dtymk protein but could not be recognized by the Dtymk specific antibody. This study expands our knowledge of nucleotide biosynthesis during zebrafish development, and also provided evidence for a compensatory TMPK-like enzyme expressed at later stages of development.

## Materials and methods

### Zebrafish embryo maintenance

Adult zebrafish of a laboratory bred population were maintained under climate controlled conditions in the Aquatic Laboratory at the Department of Biomedical Sciences and Veterinary Public Health, Swedish University of Agricultural Sciences, Uppsala, Sweden. The fish were kept at a photoperiod of 12 h light/12 h dark cycle and a water temperature of 27 °C. The fish were fed twice daily with commercial flake food Sera Vipan Nature® (Djurhobby, Uppsala, Sweden). Groups of 10 individual adult zebrafish were placed in stainless steel mesh spawning cages in 10-L tanks the day before experiments started. Spawning was initiated at onset of light the day after at 9:00 a.m. Approximately 2000 eggs were collected and assessed for fertilization under a stereo-microscope. The selected fertilized eggs were placed in Petri dishes with filtered oxygenated water (0.22 µM filter). The developing embryos were sampled at 0 (n = 60), 6 (n = 60), 24 (n = 60), 48 (n = 45), 72 (n = 30), 96 (n = 30), 120 (n = 30) and 144 (n = 30) hours post fertilization (hpf). Embryos were not fed during development. Adult zebrafish (n = 200) euthanized due to old age (3 to 4 years old of mixed gender) were also sampled. At sampling, adult fish were euthanized by decapitation after immersion in sodium bicarbonate buffered tricaine methanesulfonate solution (MS222: 500 mg/L). Dissection of body parts and organs were performed under a stereo-microscope. All experiments with zebrafish were conducted according to the ARRIVE guidelines [36].

### Protein isolation and subcellular fractionation

The nuclei, mitochondria and cytosol fractions were prepared essentially as described [30, 37]. Briefly, zebrafish embryos were washed with ice cold water at least five times before addition of protein extraction buffer (10 mM Tris-HCl pH 7.6, 25 mM KCl, 1 mM dithiothreitol (DTT), 25 mM sucrose and 1x protease inhibitor). After homogenization, the homogenates were centrifuged at 800 x g for 15 min at 4 °C to extract nuclei; the supernatant was then centrifugation at 12 000 x g for 30 min at 4 °C to isolate mitochondria. The remaining supernatant was centrifuged again two more times and the final supernatant was saved as the cytosolic fraction. The nuclei and mitochondria preparations were washed three times with the extraction buffer to avoid cytosolic protein contamination. Finally, 0.1% NP-40 was added to the nuclei and mitochondria preparations and incubated on ice for 20 min to extract the total protein. Protein concentration was determined by the Bradford method using bovine serum albumin (BSA) as standard.

### Enzyme assays

TK and TMPK activities were measured by using radiolabeled substrates essentially as described previously [30, 38] but with the reaction temperature set to 27.5 °C and the duration of the assays to 30 or 60 min.

### dNTP pool measurement

A pool of 50–75 embryos/larvae was collected at each developmental stage and homogenized immediately. Soluble nucleotides were extracted by using 60% methanol and total dNTP pools were determined essentially as described [39]. Briefly, appropriate amounts of soluble nucleotide extracts or dNTP standards were added to a reaction mixture containing 40 mM Tris/HCl, pH 7.5, 10 mM MgCl_2_, 5 mM DTT, 0.25 µM specific primed oligonucleotide, 0.75 µM ^3^ H-dTTP or ^3^ H-dATP, and 0.30 unit Taq DNA polymerase in a total volume of 20 µl. The reaction mixtures were incubated at 48 °C for 60 min, and then 15 µl of the reaction mixture were spotted onto DEAE filter paper (DEAE filtermat, PerkinElmer), and dried. The filter papers were then washed three times with 5% NaH_2_PO_4_, once with water and once with 95% ethanol. The filters were dried and the products were quantified by liquid scintillation counting (Tri-carb, PerkinElmer) after the addition of scintillation fluid (Optiphase Hisafe, PerkinElmer). The results are given as mean ± SD from 9 to 12 independent measurements.

### Anionic exchange column chromatography

Zebrafish proteins from 24 hpf embryos or 120 hfp larvae were isolated and stored at −80 °C until further analysis. Each batch provided ~ 2000–4000 fertilized embryos and three to four batches were pooled together for protein separation. A diethylaminoethanol (DEAE)-Sepharose Fast Flow (GE Healthcare) column was equilibrated with DEAE buffer (10 mM Tris/HCl pH 7.6, 25 mM KCl and 0.1 M sucrose) before the proteins were applied to the column. The flow through fraction was collected and the bound proteins were eluted with stepwise increasing KCl concentrations (50 to 250 mM) in the DEAE buffer. Fractions were collected and used to determine protein concentrations by using Bio-Rad protein assay (Bio-Rad) with BSA as standard, TK and TMPK activities determinations by using tritium labeled substrates and western blot analysis using specific antibodies.

## Western blot analysis

Polyclonal antibody against zebrafish Dtymk was designed and produced by Genscript Inc. The 13-amino acid peptide sequence chosen for immunization represented amino acid number 139–152. The corresponding sequence in human TMPK encompasses the Lid region in the human TMPK structure, which is exposed on the enzyme surface [28]. Antibody specificity was validated by ELISA (by the manufacturer) and in-house by peptide blocking experiments for western blot analysis using the 13-amino acid peptide used for immunization (data not shown). Monoclonal anti-TK1 antibody was a gift from Dr. Kiran Kumar Jagarlamudi [40]. Anti-actin antibody was purchased from Abcam. Infrared-labeled secondary antibody was from Li-COR. All western blot analyses followed standard protocol. The images shown in figures were cropped in order to improve clarity and conciseness and all original, full-length and uncropped images are provided as Additional file 1.

### Size exclusion chromatography

The Äkta Prime system was connected to a Superdex 200 10/300 GL column (GE healthcare). The column was washed extensively and equilibrated with buffer containing 10 mM Tris/HCl, pH 7.6, 100 mM NaCl, 5 mM MgCl_2_ and 5 mM DTT. Prior to loading, protein samples were filtered through a 0.22 µM filter (Merck). The flow rate was 0.3 ml/min. Fractions (0.4 ml) were collected and saved for further analysis.

### Statistical analysis

All experiments were repeated at least three times and data were analyzed with GraphPad Prism. Statistical analyses were performed by T-test, two-tailed. P > 0.05 was considered as not statistically significant, and *p < 0.05, **p < 0.01, ***p < 0.001 as significant. All data are given as mean ± standard deviation and for western blot analyses representative blots are shown.

## Supplementary Information

**Additional file 1.** Additional figures.

## Data Availability

All data generated in this study are included in the manuscript.

## Abbreviations

TMPK: Thymidylate kinase
TK: Thymidine kinase
dNTP: Deoxynucleoside triphosphate
dThd: Thymidine
dCyd: Deoxycytidine
dTMP: Thymidine 5’-minophosphate
hpf: Hours post fertilization
MW: Molecular weight
