# Supplementary material for: Differential expression of enzymes in thymidylate biosynthesis in zebrafish at different developmental stages: implications for dtymk mutation-caused neurodegenerative disorders
Source: BMC Neurosci. 2022 Mar 27;23:19. doi: 10.1186/s12868-022-00704-0 (PMC8962455; doi:10.1186/s12868-022-00704-0)
Supplement: Supplementary file 1 — Additional file 1. Additional figures. [file 12868_2022_704_MOESM1_ESM.pptx]

## Slide 1
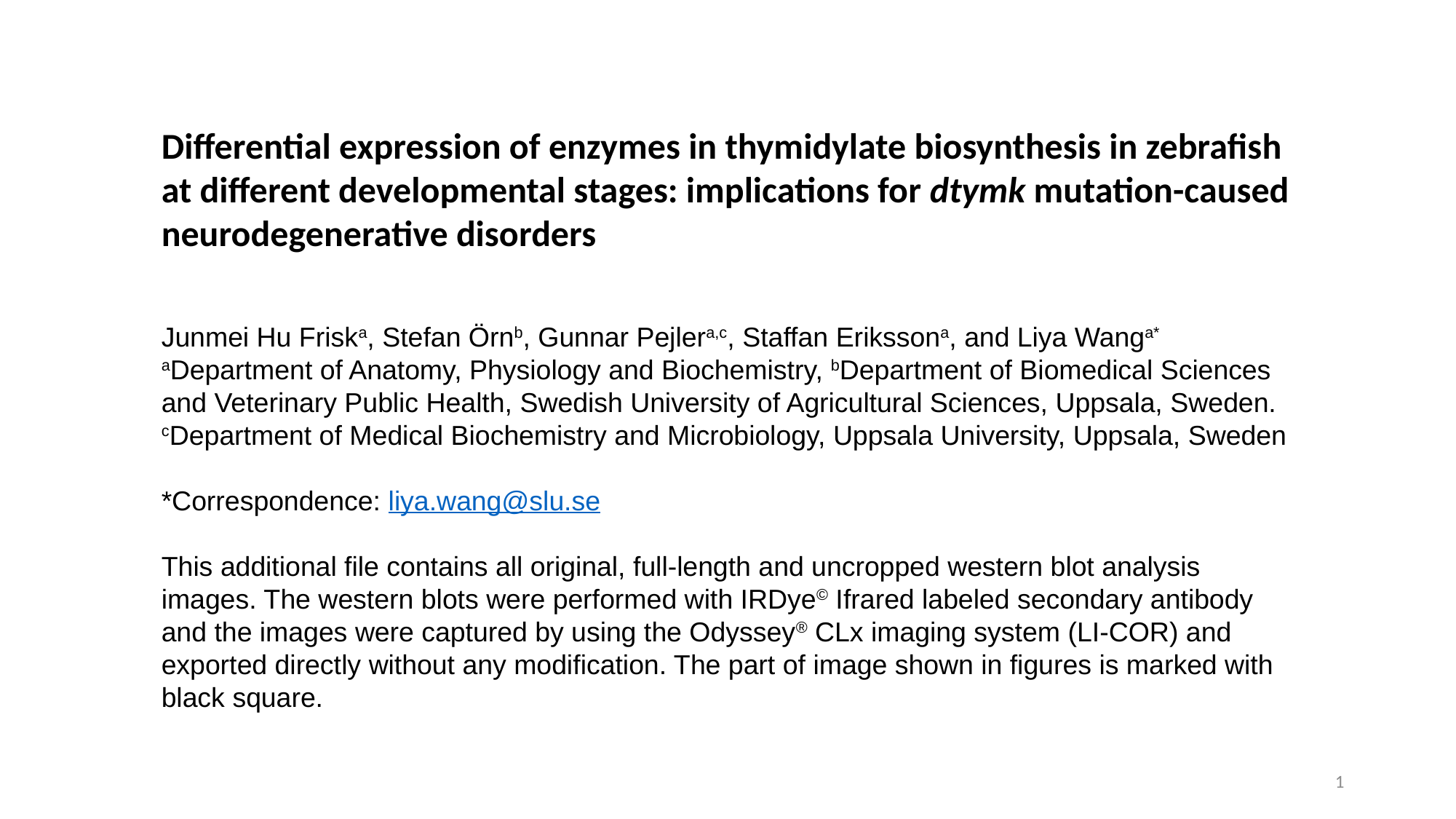

Differential expression of enzymes in thymidylate biosynthesis in zebrafish at different developmental stages: implications for dtymk mutation-caused neurodegenerative disorders
Junmei Hu Friska, Stefan Örnb, Gunnar Pejlera,c, Staffan Erikssona, and Liya Wanga*
aDepartment of Anatomy, Physiology and Biochemistry, bDepartment of Biomedical Sciences and Veterinary Public Health, Swedish University of Agricultural Sciences, Uppsala, Sweden.
cDepartment of Medical Biochemistry and Microbiology, Uppsala University, Uppsala, Sweden
*Correspondence: liya.wang@slu.se
This additional file contains all original, full-length and uncropped western blot analysis images. The western blots were performed with IRDye© Ifrared labeled secondary antibody and the images were captured by using the Odyssey® CLx imaging system (LI-COR) and exported directly without any modification. The part of image shown in figures is marked with black square.
1

## Slide 2
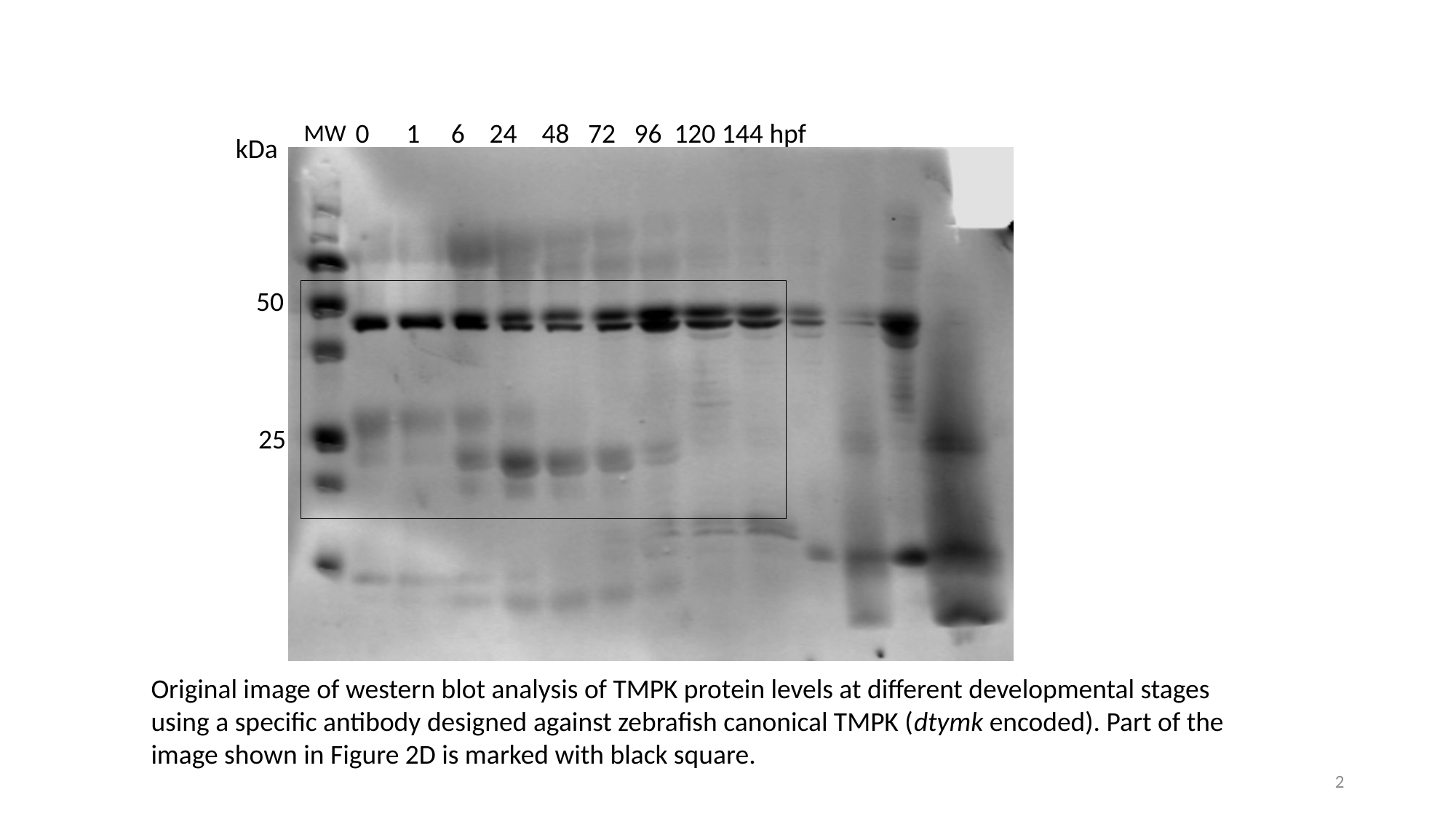

0 1 6 24 48 72 96 120 144 hpf
MW
kDa
50
25
Original image of western blot analysis of TMPK protein levels at different developmental stages using a specific antibody designed against zebrafish canonical TMPK (dtymk encoded). Part of the image shown in Figure 2D is marked with black square.
2

## Slide 3
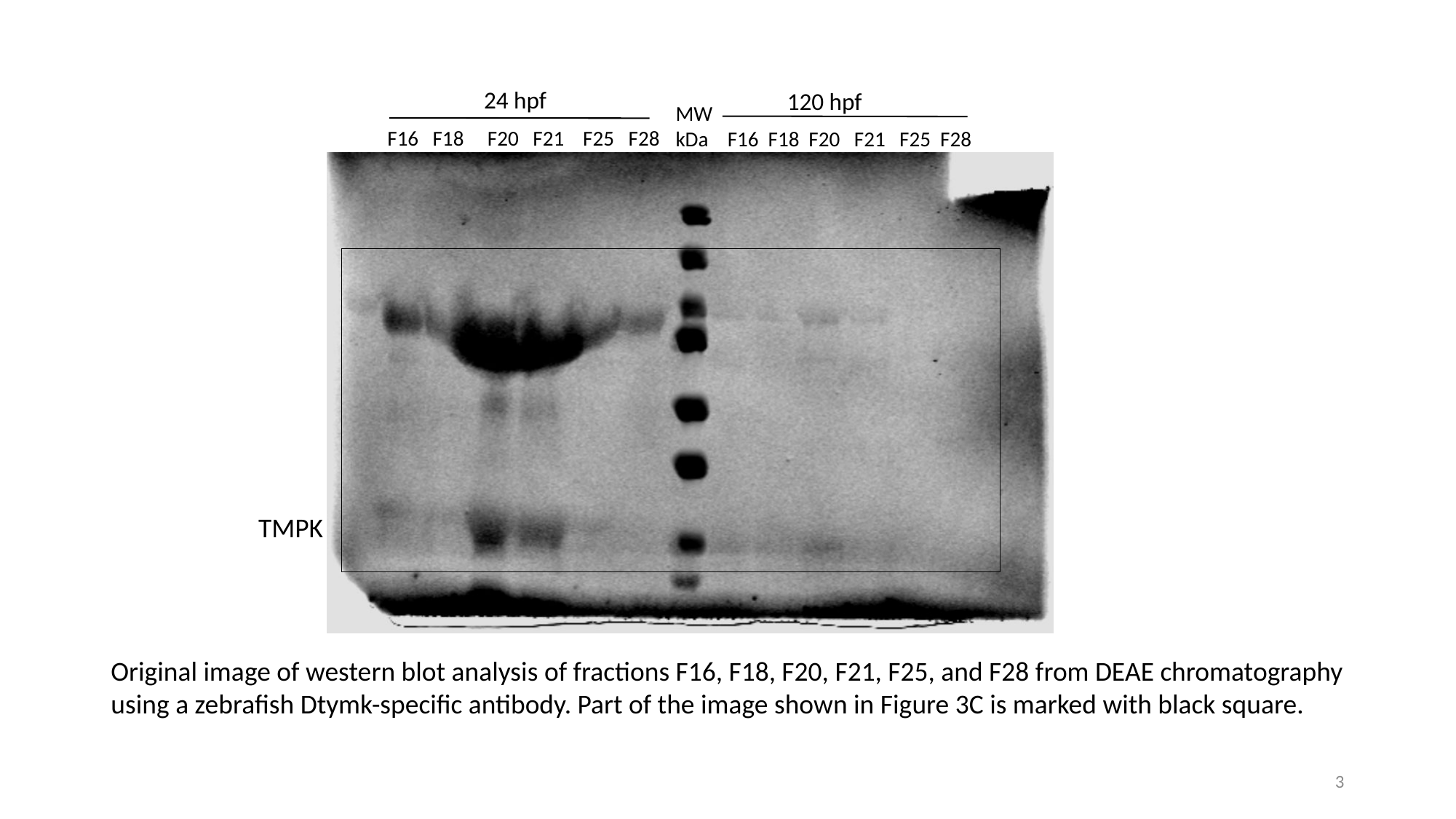

24 hpf
120 hpf
MW
kDa
F16 F18 F20 F21 F25 F28
F16 F18 F20 F21 F25 F28
TMPK
Original image of western blot analysis of fractions F16, F18, F20, F21, F25, and F28 from DEAE chromatography using a zebrafish Dtymk-specific antibody. Part of the image shown in Figure 3C is marked with black square.
3

## Slide 4
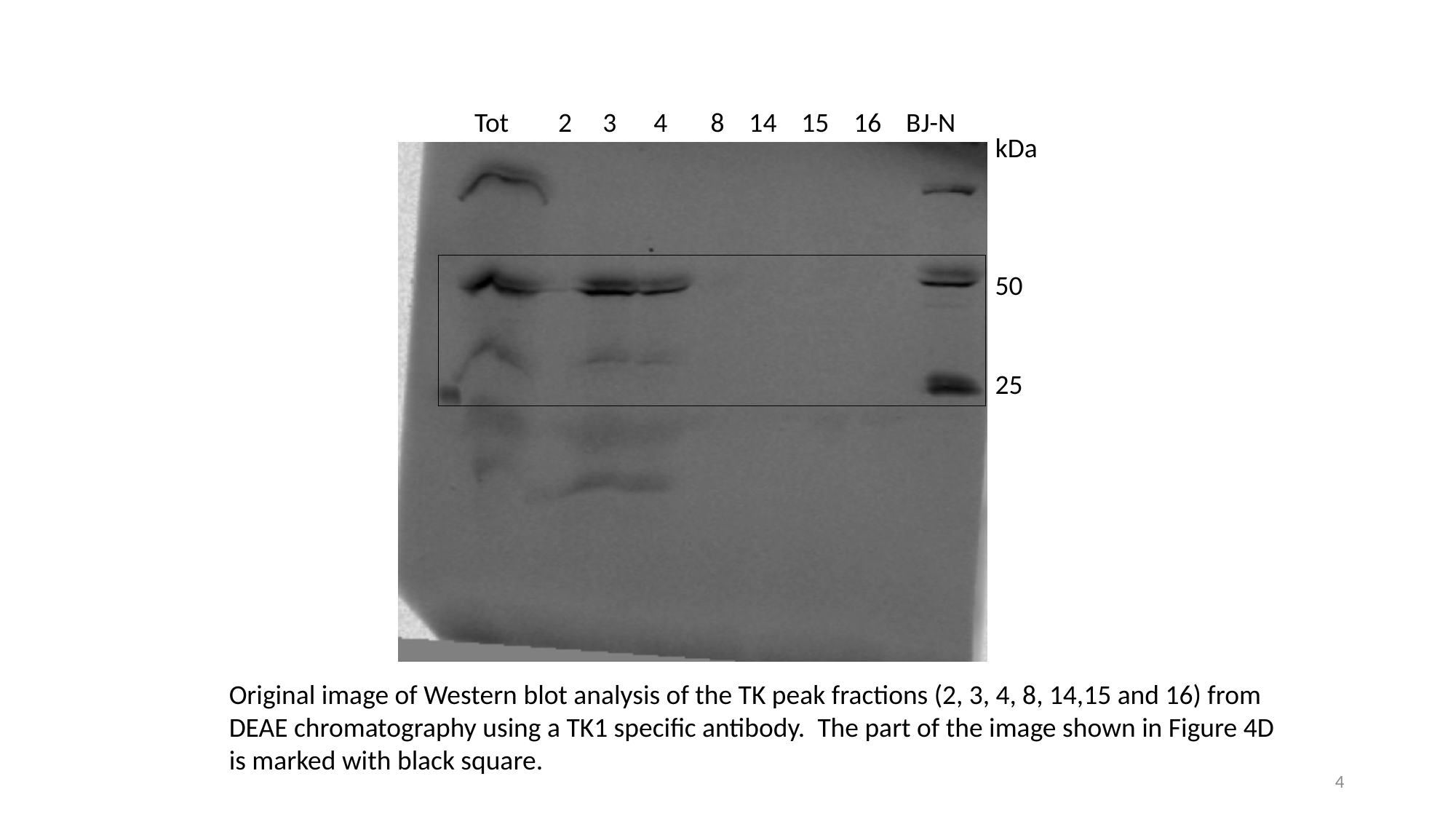

Tot 2 3 4 8 14 15 16 BJ-N
kDa
50
25
Original image of Western blot analysis of the TK peak fractions (2, 3, 4, 8, 14,15 and 16) from DEAE chromatography using a TK1 specific antibody. The part of the image shown in Figure 4D is marked with black square.
4

## Slide 5
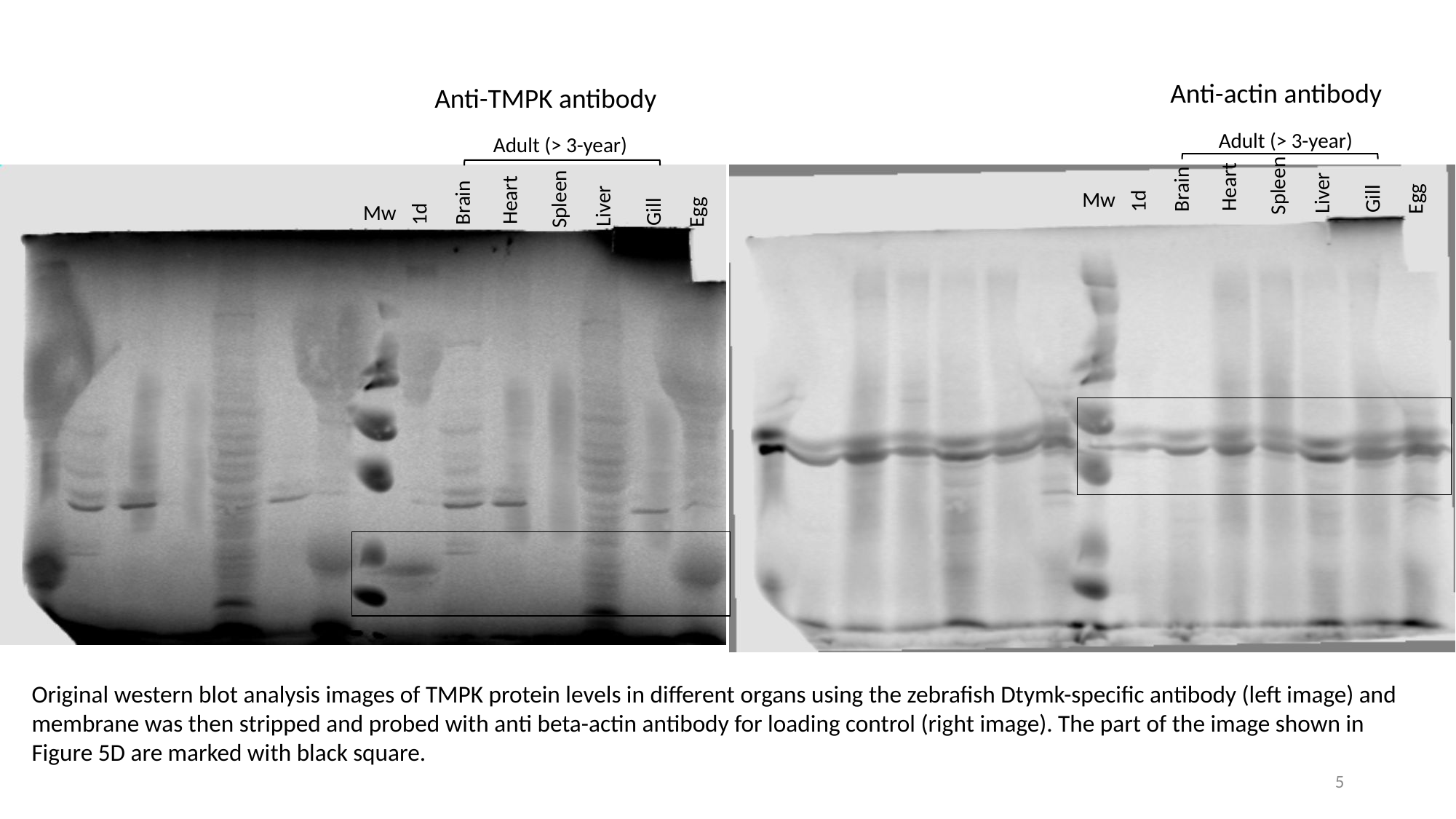

Anti-actin antibody
Anti-TMPK antibody
Adult (> 3-year)
Adult (> 3-year)
Spleen
Heart
Brain
Liver
Gill
Mw
Egg
1d
Spleen
Heart
Brain
Liver
Gill
Mw
Egg
1d
Original western blot analysis images of TMPK protein levels in different organs using the zebrafish Dtymk-specific antibody (left image) and membrane was then stripped and probed with anti beta-actin antibody for loading control (right image). The part of the image shown in Figure 5D are marked with black square.
5
